# Scurvy

**Published:** 1889-05

**Authors:** Frederick P. Henry

**Affiliations:** Philadelphia. Physician to the Philadelphia and Jefferson College Hospitals


### Scurvy.

BY FREDERICK P. HENRY, M.D., PHILADELPHIA. PHYSICIAN TO THE PHILADELPHIA AND JEFFERSON COLLEGE HOSPITALS.

[Delivered at the Philadelphia Hospital March 16, 1889.]

At the present day examples of this disease are rarely seen, and there can be no doubt that its frequency even in former times was much overrated. In the older medical works the term "scorbutic" was very loosely employed, and the same criticism applies to popular works of fiction of the early part of this century. The writers of these books doubtless obtained their notions concerning this subject from the current medical literature. Readers of Dickens will surely recall the "scorbutic youth" who made himself so conspicuously disagreeable at Bob Sawyer's celebrated supper, and whose countenance was probably the seat of an eruption of acne. This and other skin eruptions were at that time supposed to be of a scorbutic nature; an opinion which is without scientific foundation.

I believe that I am acquainted with the entire literature of this subject for 1888, and it is exceedingly scanty. Besides three cases which I made the subject of a clinical lecture, published in the *Medical and Surgical Reporter*, June 16, 1888, two have been reported by Variot[1] and one by Barkas.[2] An elaborate article on the "Occurrence of Scurvy among Troops and its Prevention," appeared in the *Practitioner* for May of the same year. In the course of it, its author, J. Hickman, remarks that the occurrence

1 Le Bulletin Med., September 19, 1888.
2 Australian Med. Gaz., January 15, 1888.

of a single case among troops "shows negligence, and is a reproach to the authorities." I cannot accept this statement without a word of explanation. A soldier, a prisoner, an inmate of an almshouse, or a hospital patient may be supplied with anti-scorbutic articles of food, and yet develop scurvy, because he does not eat them. Quite recently I was called to attend a rapidly growing boy, sixteen years old, who had gradually become weak and anæmic, and whose gums, on examination, I found to be decidedly tender and spongy. He had a comfortable home and was supplied with an abundance of nutritious food, but, as his mother told me in reply to my questions, he lived almost solely upon bread and meat.

The two patients I bring before you this morning owe their disease to the fact that they have steadily refused to eat potatoes, although abundantly supplied with them. The potato is one of the best of anti-scorbutics, aud is believed to derive this property from the potassium which it contains in large amount. The opinion that potassium is a highly anti-scorbutic substance, and that its absence from the food is one of the causes of scurvy, was first announced about forty years ago, by Garrod, and was addpted by Liebig.

The diagnosis of a case of primary scurvy presents no difficulty to one who has closely observed a single well-marked case of the disease; but secondary cases, *i. e.*, cases occurring in the course of another disease, are very apt to be overlooked. The patient being already confined to bed, the pains on walking, so great in the primary forms, are unperceived, while the increasing feebleness is attributed to the primary affection. Lasegue and Legroux, who studied nearly one hundred cases of scurvy in the central infirmary of the prisons of Paris, during the siege of that city by the Germans in 1870, state with reference to their cases of secondary scurvy, that many of them would have been overlooked but for their daily careful search for its manifestations in *all the patients* under their care. The patients before you did not present themselves for treatment, but were recognized as cases of scurvy by one of the resident physicians, who systematically went through the "out wards" looking for signs of scurvy. The symptoms are well marked in both cases. In one, the gums at the time of admission were swollen to such an extent as almost to conceal the incisor teeth. In the other, the affection of the gums, though well marked, is not so extreme as in the first case. In both, as you observe, there are large subcutaneous extravasations of blood on the anterior surface of both tibiæ.

The influence of the mind over the body is often referred to in general terms, but seldom with special reference to the nutritive functions. Depressing emotions, whether by direct nervous action upon those functions, or indirectly by impairing the appetite, certainly predispose to scurvy. It is even believed by Barkas that the prevalence of scurvy among the Australian shepherds is in part due to their monotonous solitary life. In their case, however, the diet, in his opinion, is all-sufficient to account for it, for it is composed "solely of tea, salt beef, and 'damper.'" It is scarcely necessary to say that the emotions of an "out-warder" are depressing, for the very fact of his entrance here announces that he has abandoned hope.

The treatment of these cases will be almost exclusively dietetic. They will be supplied with onions, potatoes and lemon juice—the best of anti-scorbutics. I can say this much with reference to their treatment, but no more, since neither of them is under my care. I am indebted to Drs. Tyson and Walker, in whose wards they belong, for the opportunity of showing them to you this morning.

They are both anæmic, and in one the precise degree of anæmia has been ascertained by Dr. Hamill, who examined the blood with Gower's instruments. The corpuscles numbered about 4,000,000 per cubic millimeter (80 per cent. of the normal), and the coloring matter was reduced to 60 per cent. of the normal. Chalybeates are therefore indicated, and an excellent mixture in such cases is one containing the tincture of the chloride of iron and the fluid extract of ergot.

